# An Enormous Inguinal Hernia

**Published:** 1889-10

**Authors:** L. G. Hardman

**Affiliations:** Harmony Grove, Ga.


*The Atlanta Medical and Surgical Journal, Atlanta, Ga.:*

I enclose a photograph of a patient of mine who has one of the largest inguinal hernias I have ever seen. He is now fifty-five years of age and is in the best of health otherwise. He has never suffered any inconvenience from the hernia, except that arising from its weight and size. It began thirty-four years ago after a severe strain, and continued to increase gradually in size. It was easily reduced until seven years ago. Since that time no systematic attempt has been made to reduce it. The tumor measures twenty-three inches in circumference and fifteen inches in length. Notwithstanding this he works daily in the field and enjoys perfect health. The feel and percussion seem to indicate an entero-epiplocele.

L. G. HARDMAN, M. D.

